# Murine Bone Marrow Niches from Hematopoietic Stem Cells to B Cells

**DOI:** 10.3390/ijms19082353

**Published:** 2018-08-10

**Authors:** Michel Aurrand-Lions, Stéphane J. C. Mancini

**Affiliations:** Aix Marseille University, CNRS, INSERM, Institut Paoli-Calmettes, CRCM, 13009 Marseille, France

**Keywords:** early hematopoiesis, B lymphopoiesis, bone marrow niches, stromal cells

## Abstract

After birth, the development of hematopoietic cells occurs in the bone marrow. Hematopoietic differentiation is finely tuned by cell-intrinsic mechanisms and lineage-specific transcription factors. However, it is now clear that the bone marrow microenvironment plays an essential role in the maintenance of hematopoietic stem cells (HSC) and their differentiation into more mature lineages. Mesenchymal and endothelial cells contribute to a protective microenvironment called hematopoietic niches that secrete specific factors and establish a direct contact with developing hematopoietic cells. A number of recent studies have addressed in mouse models the specific molecular events that are involved in the cellular crosstalk between hematopoietic subsets and their niches. This has led to the concept that hematopoietic differentiation and commitment towards a given hematopoietic pathway is a dynamic process controlled at least partially by the bone marrow microenvironment. In this review, we discuss the evolving view of murine hematopoietic–stromal cell crosstalk that is involved in HSC maintenance and commitment towards B cell differentiation.

## 1. Introduction on Early Hematopoiesis and B Lymphopoiesis

In mammals, adult hematopoiesis occurs in the bone marrow (BM). Deciphering the different stages of hematopoiesis and the developmental cues driving stem cell commitment towards a particular lineage is essential in regenerative medicine and in the development of treatments for hematopoietic diseases. Lymphoid development from hematopoietic stem cells (HSC) has been extensively dissected in mouse models through genetic ablation of key genes and phenotypic characterization of cell subsets at different maturation steps.

**Figure 1:**
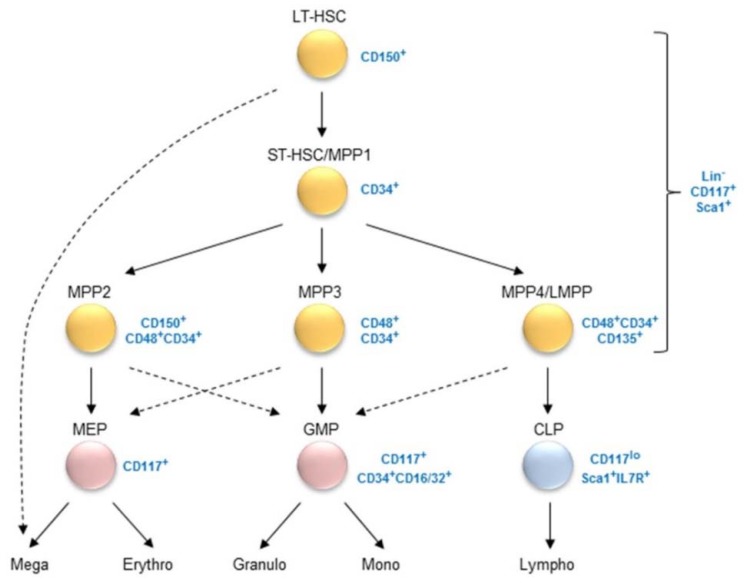
Murine early hematopoiesis from long-term hematopoietic stem cells towards the different lineages. Plain and dashed arrows show the main and the alternative branch points, respectively. Hematopoietic progenitors do not express markers of the different hematopoietic lineages and are therefore said to be lineage-negative (Lin^−^). Markers used to characterize the different hematopoietic progenitors are indicated. LT-HSC: long-term hematopoietic stem cell (HSC); ST-HSC: short-term HSC; MPP: multipotent progenitor; LMPP: lymphoid-biased multipotent progenitor; MEP: megakaryocyte-erythroid progenitor; GMP: granulocyte-monocyte progenitor; CLP: common lymphoid progenitor; Mega: megakaryocyte; Erythro: erythrocyte; Granulo: granulocyte; Mono: monocyte; Lympho: lymphocyte; Lin: lineage markers.

HSC have the lifelong capacity to self-renew and to give rise to all hematopoietic lineages. The existence of HSC was first demonstrated by Till, McCulloch, and colleagues, who reported that the bone marrow contains cells having the capacity to reconstitute lethally irradiated mice and form myelo-erythroid colonies in the spleen (Colony-Forming Unite Spleen, CFU-S) [1,2]. Later, Weissman and colleagues strongly contributed to the phenotypic identification of hematopoietic cells enriched for HSC in mouse. The multipotent and self-renewal potentials were first shown to be properties of a subset of cells lacking markers of committed hematopoietic lineages—so-called lineage-negative (Lin^−^)—and expressing Sca1 and low levels of Thy1.1 [3]. It was then demonstrated that the Lin^−^CD117^+^Sca1^+^ (LSK) fraction retained the multipotent potential and could be further fractioned into long-term (LT) and short-term (ST) repopulating subsets [4,5]. The acquisition of CD34 expression by murine HSC marks the transition from LT- to ST-HSC [6]. Later on, the introduction of differentially expressed markers such as CD135 (Flk2/Flt3) and signaling lymphocytic activation molecule (SLAM) family proteins led to the identification of different multipotent progenitor (MPP) subsets: the LSK CD150^+^CD48^−^CD34^−^ subset LT-HSC on top of the hierarchy and more differentiated MPPs with a full spectrum of lineage reconstitution (ST-HSC/MPP1) or with a biased (but not definitive) engagement towards particular lineages (MPP2 to MPP4; Figure 1) [6,7,8,9,10,11,12]. Finally, advances in single-cell technology demonstrated that the phenotypic boundaries between subsets are not strict, but rather represent a continuum of progenitor states acquiring lineage restrictions progressively. This is usually illustrated as a landscape of hills and valleys branching from LT-HSC up to the different committed lineages [13,14,15,16,17,18].

**Figure 2:**
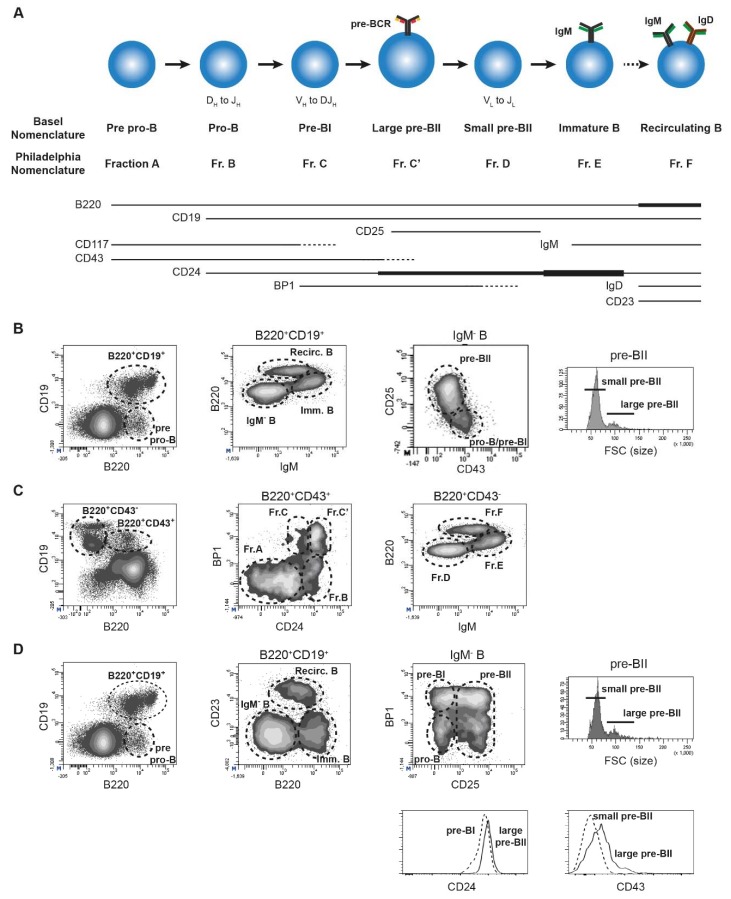
Murine bone marrow (BM) B cell differentiation. (**A**) Schematic representation of the different B cell differentiation stages with their denomination according to the Basel and Philadelphia nomenclatures. The pattern of expression of the main markers used to characterize each subset is shown with a line. The thickness of the line is representative of the level of expression. The dotted lines indicate subsets where expression is progressively lost. VDJ_H_ and VJ_L_ rearrangements are indicated; (**B**) BM B lymphopoiesis analysis by flow cytometry according to the Basel nomenclature; (**C**) BM B lymphopoiesis analysis by flow cytometry according to the Philadelphia nomenclature. The gating strategy and the main subsets are indicated in the panels; (**D**) BM B lymphopoiesis analysis by flow cytometry taking into consideration both nomenclatures. This strategy improves the resolution of the subsets, particularly of the pro-B, the pre-BI and the large pre-BII fractions (Fr. B to Fr. C’). Indeed, while the separation between pro-B and pre-BI cells (Fr. B and C) is not possible with the Basel nomenclature and the distinction between pre-BI and large pre-BII (Fr. C and C’) with CD24 is dependent on mouse strains, the simultaneous use of BP1 and CD25 allows a clear definition of the three subsets.

Progression from MPP4/LMPP to the common lymphoid progenitor (CLP) subset marks the entry into the lymphoid lineage and is characterized by interleukin-7 receptor (IL7R) upregulation [9,19]. While CLPs (CD117^lo^Sca1^+^IL7R^+^) have the capacity to differentiate into all lymphoid subsets, the upregulation of Ly6D marks the engagement into the B cell lineage [20]. The low natural killer and T cell potentials retained by the Ly6D^+^ CLPs (called B lymphoid progenitors, BLP) are lost upon entry in the earliest pre-pro-B cell stage, as indicated by B220 expression. The following B cell differentiation steps have a specific and crucial role in the acquisition of a non-autoreactive B cell receptor (BCR, or immunoglobulin, Ig) repertoire which is essential for efficient adaptive immune responses. More than two decades ago, both Hardy and Rolink established independent combinations of markers, known as the Philadelphia and Basel nomenclatures, respectively, which are still standards for the phenotypic characterization of the different BM B cell subsets in mice (Figure 2A) [21,22]. Both nomenclatures use B220 and CD19 as lineage markers as well as the surface expression of BCR as a marker of maturity. Hardy and colleagues established their classification based on CD43, CD24, and BP1 expression, while Rolink and colleagues used CD43, CD117, and CD25 [23,24]. Importantly, recent advances in multi-parameter flow cytometry now give the opportunity to consider both strategies together and improve the resolution of each subset (Figure 2B–D). These results clearly show that both phenotypic strategies of B cell maturation intermediates are valid and overlapping.

B cell specification initiates at the pre-pro-B stage and is driven by the E2A transcription factor [25]. However, the definitive commitment to the B cell lineage occurs at the pro-B cell stage and is controlled by Pax5. Indeed, loss of Pax5 results in B cell differentiation arrest at the pro-B cell stage, and *Pax5*^−/−^ pro-B cells acquire the capacity to differentiate into other lymphoid and myeloid lineages [26,27,28]. The rearrangements of genes encoding the Ig heavy chain (IgH) are initiated at the pro-B cell stage between the D_H_ and J_H_ segments, while the complete VDJ_H_ recombination takes place at the pre-BI stage. If a functional Igμ protein is generated from the recombination product, it is associated with the surrogate light chain (SLC), composed of the invariant λ5 and VpreB proteins, and with the Igα/Igβ signaling complex to form the pre-BCR [29,30,31,32]. Pre-BCR expression by large pre-BII cells induces proliferation and differentiation towards the small pre-BII stage, where pre-BCR expression is downmodulated, and Ig light chain (IgL) gene rearrangements are initiated. Upon expression of a functional IgL chain, the BCR is formed by association with the Igμ chain at the immature B cell stage. Finally, immature B cells expressing an autoreactive BCR receive a ‘no-go’ signal and have the possibility of reinitiating rearrangements through receptor editing [33], while immature B cells expressing a non-autoreactive BCR leave the BM to complete their maturation in the periphery.

Self-renewal, differentiation, and commitment events occurring during hematopoietic progenitor development as well as B lymphopoiesis are driven by complex intrinsic regulatory networks described in details elsewhere [25,34,35]. However, these networks are triggered and regulated by extrinsic signals delivered by cells of the BM microenvironment through the secretion of growth factors and direct cellular interactions. These supportive regions, called niches, are composed of mesenchymal, endothelial, and hematopoietic cells. Advances in the analysis of stromal cell niches in vivo have been possible thanks to the development of reporter and tissue-specific deletion systems in mouse models. In this review, we will thus focus on the known molecular mechanisms by which murine mesenchymal and endothelial cells control HSC behavior and B cell commitment.

## 2. The HSC Niche

### 2.1. Pioneer Views on the HSC Niche

The influence of cells from the BM microenvironment on the long-term growth of HSC was first demonstrated using in vitro cultures in which adherent cells were present and composed of macrophages, adipocytes, endothelial cells, and fibroblasts [36,37]. The analysis of the relationship between hematopoietic cells and their microenvironment in vivo has long been limited to the spatial localization of the different lineages in the BM conduit. The microscopic analysis of bone transversal sections following injection of tritiated thymidine suggested a centripetal differentiation of hematopoietic precursors from endosteal regions towards the center of the BM [38]. Furthermore, by measuring the progenitor self-renewal property using CFU-S assays, it was shown that the number of colonies formed was higher by transplanting cells from the sub-endosteal region rather than from the central marrow (reviewed in [39]). These results suggested that cells of the microenvironment located close to the bone border were involved in HSC maintenance.

The development of mouse models allowing tissue-specific deletion of genes or expression of fluorescent proteins as well as the technological progress in confocal microscopy have been crucial for the dissection of BM organization. The existence of an osteoblastic niche for HSC was first proposed. In a first model, the number of HSC could be significantly increased by manipulating osteoblast (OB) number through the injection of parathyroid hormone (PTH) or by specifically over-expressing the PTH receptor using the OB-specific *Col1a1* promoter [40]. Similarly, increasing spindle-shaped OB numbers through conditional deletion of the *Bmpr1a* gene in *Mx-Cre*/*Bmpr1a*^lox/lox^ mice induced a parallel increase in HSC [41]. Altogether, these results suggested the importance of an osteoblastic niche in the maintenance of HSC.

### 2.2. The Peri-Sinusoidal HSC Niche

**Figure 3:**
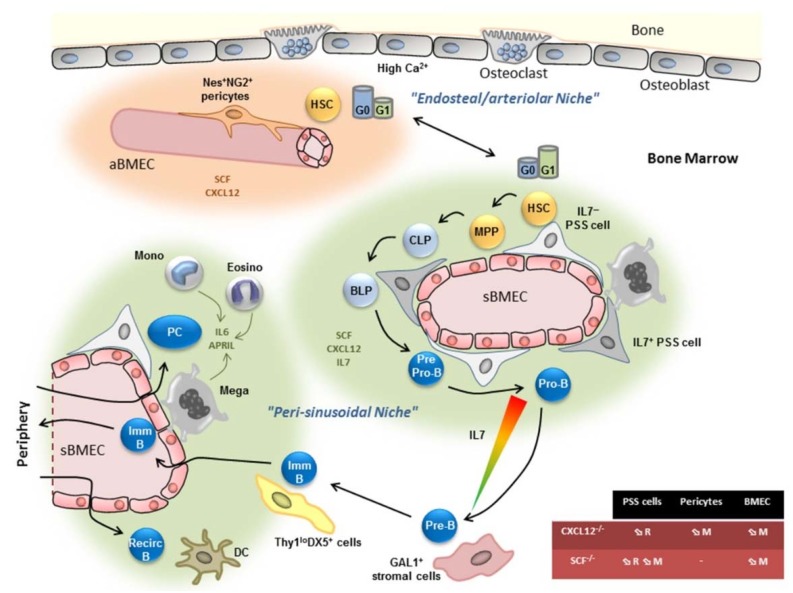
Bone marrow niches for hematopoietic stem cells and B cells. HSC are located in both endosteal/arteriolar and in peri-sinusoidal regions which express high levels of CXCL12 and stem cell factor (SCF). Quiescent HSC are enriched in the endosteal/arteriolar niche. Differentiation of MPP up to the pro-B cell stage takes place in the peri-sinusoidal niche, where the level of CXCL12 and IL7 are high. Pre-B cell then relocalize close to GAL1-expressing stromal cells located away from the sinusoids. At the next immature B cell stage, cells expressing an auto-reactive B cell receptor (BCR) are retained in the BM in order to initiate receptor editing, while non-autoreactive cells leave the BM to finish their maturation in the periphery. Mature/recirculating B cells and plasma cells follow CXCL12 gradients to home to the BM. Recirculating B cell survival relies on dendritic cells. PC survival relies on the secretion of IL6 and A proliferation-inducing ligand (APRIL) by monocytes, eosinophil, and megakaryocytes. The colored triangle represents the gradient of IL7 expression from high (red) to low (green). The table in the bottom right summarizes the influence of CXCL12 and SCF specific deletion in PSS cells, pericytes, or BMEC on HSC retention (R) and maintenance (M). MPP; multipotent progenitor; CLP: common lymphoid progenitor; BLP: B lymphoid progenitor; Imm. B: immature B cell; Recirc. B: recirculating B cell; PC: plasma cell; Mono: monocyte; Eosino: eosinophil; Mega: megakaryocyte; DC: dendritic cell; aBMEC: arteriolar bone marrow endothelial cell; sBMEC: sinusoidal BMEC; PSS cell: peri-sinusoidal stromal cell.

The existence of an osteoblastic niche was then challenged by the development of a mouse model in which green fluorescent protein (GFP) was knocked-in downstream of the C–X–C Motif Chemokine Ligand 12 (CXCL12) promoter that allowed visualization of CXCL12-producing cells [42,43]. CXCL12 was first described as a growth factor for early B cells, but deletion of CXCR4, its receptor, also induced major hematopoietic defects [44,45]. Deletion of *Cxcr4* in adult HSC using *Mx-Cre*/*Cxcr4*^lox/−^ mice injected with poly(I:C) induced a strong decrease in HSC number, suggesting a crucial role of CXCL12 in HSC maintenance [43]. However, expansion of the HSC population was observed when *ROSA-Cre ERT2* and Tamoxifen were used to delete *Cxcr4*, suggesting that HSC maintenance and expansion are tightly dependent on the chemokine context of the bone marrow [46]. CXCL12 was further shown to be a chemoattractant for human and mouse hematopoietic progenitors and to allow their retention in the BM [47,48,49]. In vivo tracing of CXCL12-expressing cells using *Cxcl12-GFP* or *Cxcl12-dsRed* knock-in mice showed that GFP was strongly expressed by reticular cells (called CAR cells for CXCL12-abundant reticular cells), which were scattered throughout the BM and in contact with the vasculature. In contrast, the expression of *Cxcl12* by BM endothelial cells (BMEC) and OB was 100 and 1000 times lower, respectively [42,43,50]. Accordingly, CD150^+^CD48^−^ HSC were essentially localized in peri-sinusoidal regions and in contact with CAR cells [7,43]. Specific deletion of *Cxcl12* in peri-sinusoidal stromal (PSS) cells, but not in OBs, led to an increase in circulating HSC (Figure 3). Furthermore, specific deletion in BMEC induced a decrease in HSC frequency but no loss of retention, indicating that CXCL12 plays a differential role in BMEC and PSS cells by allowing HSC maintenance and retention, respectively [50,51,52]. Stem cell factor (SCF), the ligand of the receptor tyrosine kinase c-kit, was also shown to be implicated in stem cell maintenance [53]. The use of *SCF-GFP* knock-in and *SCF-GFP*/*CXCL12-dsRed* double knock-in mice showed that SCF is expressed by BMEC and co-expressed with CXCL12 by PSS cells [50,54]. Specific deletion of *Kitl* encoding SCF in PSS cells decreased HSC maintenance and retention. In contrast, deletion in BMEC or OB resulted, respectively, in decreased HSC maintenance or in an absence of phenotype (Figure 3 [54]). Altogether, these results cast doubt over the existence of an osteoblastic niche and demonstrate the importance of perivascular niches, and more particularly of PSS cells, for HSC maintenance and retention.

In light of the recent knowledge accumulated on mesenchymal cell niches and development, it seems likely that the parallel increase in OB and HSC numbers is only correlative and that HSC are instead regulated by an osteoblastic progenitor. Indeed, in vitro differentiation assays have shown that CAR and PSS cells have the capacity to differentiate into osteoblasts or adipocytes [55,56]. Furthermore, PTH/PTHR signaling, which was shown to increase OB number, is able to directly stimulate PSS cell number and to favor differentiation into OB [40,56]. Finally, inducible and non-inducible lineage-tracing mouse models confirmed that PSS cells contain progenitors of osteoblasts in adult BM [57,58].

### 2.3. The Endosteal/Peri-Arteriolar Niche

Despite the clear involvement of peri-sinusoidal niches in HSC maintenance, some results still argue in favor of a function for the endosteal niche. Indeed, because of bone remodeling activity, the local concentration of calcium at the endosteal surface is high. Interestingly, HSC, which express the calcium-sensing receptor (CaSR), were found to be strongly decreased in *Casr*^−/−^ mice. In addition, transplanted *Casr*^−/−^ HSC, unlike wild-type (WT) HSC, failed to localize close to the endothelium [59]. The adhesion to extra-cellular matrix proteins as well as the migration towards CXCL12 of HSC treated in vitro with a CaSR agonist were increased. Homing and engraftment into the BM of in vitro treated HSC were also improved [60]. Finally, the frequency of quiescent HSC was reported to be higher close to the bone surface in endosteal and trabecular regions [61], indicating that these regions may indeed play a role in HSC maintenance.

Importantly, endosteal and trabecular regions are enriched in arteries, while sinusoidal venous structures are more central [62,63]. By taking into consideration the large extent of the sinusoidal network in the BM compared to arteriolar structures, Frenette and colleagues showed that the proportion of HSC in the vicinity of arterioles was highly significant [62]. Furthermore, quiescent HSC identified by their capacity to retain EdU (5-ethynyl-2′-deoxyuridine) labelling in the long term, by their low levels of reactive oxygen species (ROS) or by their high expression of hypoxia-inducible transcription factor 1 (HIF-1α) [6,64,65], were found to be frequently located close to arteriolar cells [62,66,67]. Finally, low vessel permeability ensures HSC quiescence and retention in the BM [67]. Indeed, sinusoidal BMEC (sBMEC), but not arteriolar BMEC (aBMEC), were shown to be permeable and to be the site of trans-endothelial trafficking of HSPC. Notably, when the integrity of aBMEC was affected, HSC and HSPC numbers were decreased, while HSPC trafficking was increased, confirming the importance of arteriolar niches for HSC quiescence. Also, NG2^+^Nestin^+^ pericytes associated with arterioles were shown to express HSC niche genes, including CXCL12 [51,62]. These cells were mainly located at the metaphysis and adjacent to cortical bone at the diaphysis [67]. When pericytes were specifically depleted upon injection of tamoxifen and diphtheria toxin in *NG2-CreERT*/*ROSA26iDTR* mice (iDTR: inducible Diphteria Toxin Receptor), HSC were decreased and less quiescent and relocalized away from arteries [62]. A similar effect was observed upon the specific deletion of CXCL12, but not of SCF, from pericytes [51].

Altogether, these results suggest that cells of the endosteal/peri-arteriolar region control HSC maintenance and quiescence, while cells present in the peri-sinusoidal region control HSC maintenance and retention (Figure 3). CXCL12 expressed by PSS cells, pericytes, and BMEC as well as SCF expressed by PSS cells and BMEC play a crucial role in these functions, but other factors have also been identified. Furthermore, the complexity of the HSC niche is not limited to mesenchymal cells and BMEC, as the involvement of hematopoietic cells (megakaryocytes and macrophages) and Schwann cells have also been demonstrated. The function of these cells in the maintenance of HSC homeostasis has been extensively discussed elsewhere [68,69].

## 3. Niches for Lymphoid Progenitors

The localization of HSPCs was first performed by tracking injected stained cells using bi-photon live imaging [70]. While HSC were found close to the bone surface, MPPs and more differentiated progenitors were found further away, but their association to particular regions was not studied. Because of the strong influence of CXCL12 on HSC maintenance, it has been difficult to assess its specific role on the development of more committed progenitors. In contrast, the function of IL7 in the differentiation of BLPs, but not of CLPs, has been clearly established using *Il7*^−/−^ mice [71]. Contradictory results have been obtained when *Cxcl12* and *Il7* were specifically deleted in OBs. Indeed, CLP number decreased upon *Cxcl12* deletion using the *Col2.3-Cre* but not the *Bglap-Cre* systems, although both promoters were supposed to be expressed in OBs [50,52]. Conversely, CLPs were decreased upon *Il7* deletion using the *Bglap-Cre*, but not the *Col2.3-Cre*, system [72,73]. Of note, both promoters drive partial Cre expression in perivascular stromal cells of the BM [54,74]. Therefore, it can be speculated that CLPs are controlled by stromal cells located away from the endosteum.

We previously demonstrated using the *Il7-Cre*/*Rosa-eYFP* mouse model and by qPCR on sorted cells, that PSS cells expressing CXCL12 were the main source of IL7 [75]. A more recent study confirmed our results by suggesting the existence of IL7^−^ and IL7^+^ PSS cells [73]. Importantly, BMEC, but not OBs, express low levels of IL7, confirming that the phenotype observed upon *Il7* deletion using *Bglap-Cre* or *Col2.3-Cre* could be attributed to Cre expression in perivascular stromal cells. Specific deletion of *Cxcr4* in MPPs or CLPs showed the crucial role played by CXCL12 on MPP development and on the BLP fraction of CLP [73]. Furthermore, CXCL12 was shown to position BLPs close to IL7^+^ PSS cells in order to get an efficient stimulation through the IL7R signaling pathway. Specific deletion of *Il7* in PSS cells using the *LepR-Cre* system induced a specific decrease in BLPs, confirming the phenotype observed in *Il7*^−/−^ mice [71,73]. Finally, HSC and MPPs were found indifferently in contact with both IL7^−^ and IL7^+^ PSS cells [73]. However, it cannot be excluded that these subsets may have differential functions. Indeed, while *Cxcl12* specific deletion in all PSS cells using the *LepR-Cre* system induced a loss of retention of HSC, specific deletion in IL7^+^ PSS cells using *Il7-Cre* mice had only a marginal effect on HSC and MPP numbers and no effect on HSC retention [50,51,73]. Furthermore, specific deletion of *Kitl* (SCF) in all PSS cells induced a decrease in HSC maintenance and retention, but only a decrease in HSC maintenance when deleted in IL7^+^ PSS cells. Therefore, the specific function of the different PSS subsets remains to be addressed.

Taking into consideration the most recent advances in niche biology, HSC, MPP, and CLP subsets are most likely preferentially located in peri-sinusoidal regions (Figure 3). These different data are in agreement with the hemosphere model proposed by Adams and colleagues, in which clonal hematopoiesis was observed in micro-anatomical structures composed of stromal and endothelial cells [76].

## 4. B Cell Niches

### 4.1. Pioneer Views on B Cell Niches

Early on, Whitlock and Witte demonstrated that adherent BM cells sustain long-term B cell cultures [77]. By removing glucocorticoids, known to impair lymphocyte development, from Dexter type cultures, differentiation of B cells, including IgM^+^ immature B cells, could be observed for more than 10 weeks, and the recovered cells could be clonally expanded. Stromal cell lines were then derived from these cultures, leading to the identification of the main factors involved in early B cell development, namely, CXCL12 and IL7 [44,78,79]. The generation of cell lines with distinct phenotypic characteristics and supportive functions suggested the existence of heterogeneity between stromal cells. However, this heterogeneity may have been related to stromal cell instability, as modifications in surface marker expression could be observed upon cloning [80]. These in vitro co-culture systems were further developed by Rolink and Melchers to decipher B cell differentiation mechanisms and the influence of extrinsic cues [81,82]. They demonstrated that stromal cells and IL7 were able to maintain long-term proliferation of pro-B/pre-BI, while removal of IL7 induced their differentiation into immature B cells [81,83]. Importantly, IL7-dependent pro-B/pre-BI cell growth was inhibited by a blocking anti c-kit/CD117 antibody, indicating that c-kit, the receptor for SCF, was an important co-factor for IL7-dependent early B cell proliferation [84].

Contacts between B cells and stromal reticular cells were first observed in situ by electron microscopy on BM histological sections [85]. In this study, B cell development was proposed to be centripetal, with early B cells being mainly located close to the endosteum and in intermediate zones, while IgM^+^ B cells were positioned in the vicinity of the central sinus. Furthermore, lymphoid cells, unlike myeloid cells, were closely associated to β1 integrin-expressing reticular cells [86]. Finally, β1 integrin expression revealed heterogeneity between stromal cells, with β1-integrin^+^ cells mainly localized in the peripheral region of the BM.

### 4.2. Early B Cell Niches

The evidence for the existence of stromal cell niches for early B cell development became clear with the identification of CXCL12 and IL7 as crucial growth factors produced by the BM microenvironment. B lymphopoiesis is strongly affected in the fetal liver and in the BM of *Cxcl12*- and *Cxcr4*-deficient mice [45,87,88]. Nevertheless, because of the crucial role played by the CXCL12/CXCR4 axis in HSC and BLP [43,73,89], it is difficult to evaluate their influence on early B cell development in non-conditional KO mice. An increase in pro-B cells was however observed in the fetal blood of *Cxcr4*^−/−^ mice as well as in the blood of adult WT mice reconstituted with *Cxcr4*^−/−^ fetal liver cells, indicating a role for the CXCR4/CXCL12 axis in early B cell retention [48]. This effect was further confirmed in B cell-specific *CD19-Cre*/*CXCR4*^lox/lox^ knock-out mice [90]. Although CXCR4-deficient IgM^−^ B cells retain the capacity to proliferate in the presence of IL7 in in vitro co-cultures and to differentiate into IgM^+^ B cells in the absence of IL7, a role for CXCL12 on early B cell development cannot be excluded, since differentiation towards the immature B cell stage is impaired in the absence of CXCR4 in vivo [48,90]. Furthermore, CXCL12, together with IL7 or SCF, stimulates the proliferation of pre-B cell clones and the survival of B cell progenitors in vitro, suggesting that CXCL12 may act synergistically with other factors in the earliest steps of B cell differentiation [44,89]. Finally, CXCL12 has been shown to induce α4β1 integrin-dependent adhesion of pro-B and pre-B cells to VCAM-1 through the activation of focal adhesion kinase (FAK) [91]. Accordingly, B cell development is affected in the BM of α4 integrin- and FAK-deficient mice [92,93], and pro-B cell egress in the periphery of FAK^−/−^ mice.

As said earlier, IL7 plays an important role in pre-pro-B and pro-B cell proliferation [78,81]. Interestingly, pro-B cells need high concentrations of IL7 to proliferate, while a decrease in IL7 concentration favors differentiation toward the pre-BI stage, in which recombination between V and DJ gene segments and then intracellular expression of the Igμ chain are induced [94]. IL7 has also been implicated in B cell differentiation and survival [95,96]. As a consequence, deletion of the *Il7* and *Il7r* genes leads to a severe block at the earliest stages of B cell development [97,98]. Finally, IL7 was further shown to control B cell potential already from CLPs by regulating EBF1 and Pax5 expression [71].

The identification of the niches for early B cells is still controversial. Pre-pro-B and pro-B cells were first proposed to be associated with distinct niches, expressing, respectively, CXCL12 or IL7 [42]. However, we and others have shown that both factors are co-expressed by PSS cells [73,75]. The discrepancies between the studies may be due to the low level of IL7 expression in vivo and thus to the difficulty to detect positive cells using reporter systems or antibodies [99,100]. Therefore, one could not exclude that IL7 expression in CXCL12-expressing cells was missed in the early study by Tokoyoda and collaborators because of the lack of antibody staining sensitivity. 

Other studies have proposed that OBs may play a role in early B cell development. Acute depletion of osteoblastic cells using mice expressing the herpes virus thymidine kinase under the control of the Col1a1 promoter (*Col2.3∆-TK* Tg mice) induced a decrease in both pre-pro-B and pro-B cells. Alternatively, depletion of the G protein α subunit, using the Osx-Cre system, impaired pro-B cell development. Although the promoters used in these mouse models were first thought to be specifically activated in OBs, recent results show that they are already activated in osteo-progenitors, including PSS cells [57,58,73]. Accordingly, *Bglap-Cre*/*Cxcl12*^lox/−^ mice that lack CXCL12 expression in OBs did not show any B cell phenotype, while pre-pro-B cells were impaired in *Osx-Cre*/*Cxcl12*^lox/−^ mice. This demonstrates the importance of CXCL12 expression in osteo-progenitors for pre-pro-B cell development [52].

IL7 is essentially expressed by a subset of PSS cells and at very low levels by BMECs [73,75]. Specific depletion of IL7 from PSS cells using *LepR-Cre*/*Il7*^lox/−^ mice induced a strong decrease of B cell progenitors from the BLP stage, while depletion in BMEC using *Tie2-Cre*/*Il7*^lox/−^ mice induced a low but significant decrease only from the pro-B cell stage [73]. These results indicate that pro-B cells are more affected by low IL7 fluctuations than BLP and are consistent with the fact that pro-B cell proliferation requires high levels of IL7 [94].

Altogether, these results suggest that the niche sustaining pre-pro-B and pro-B cell homeostasis is located in the peri-sinusoidal region, where both CXCL12 and IL7 levels are high (Figure 3). However, its precise location and the nature of the stromal cells involved in the control of their development remain to be defined.

### 4.3. The Pre-B Cell Niche

The expression of the pre-BCR at the large pre-BII stage is a crucial checkpoint allowing the selection of functional Igμ chains, the amplification of cells expressing such chains—ensuring higher Ig diversity—, and the induction of IgL recombination. The SLC plays a crucial role in pre-BCR signaling. Indeed, deletion of either λ5 or VpreB leads to a severe block at the pre-BI/large pre-BII transition, and deletion of both results in a complete block of differentiation [101,102,103]. Pre-BCR signaling relies on ligand-independent and ligand-dependent mechanisms, which both implicate the extra loop (EL) of λ5. Interactions between adjacent λ5-EL and VpreB-EL or between λ5-EL and a glycosylated chain linked to the Igμ constant region at position N46 were proposed to induce self-aggregation of the pre-BCR, resulting in tonic signaling [104,105]. This tonic signal is, however, increased by inducing pre-BCR cross-linking with an anti IgM antibody, suggesting the existence of a ligand for the pre-BCR [104]. Heparan sulfate proteoglycans present at the surface of stromal cells have the capacity to bind λ5-EL [106]. Interestingly, pretreatment of pre-B cells with heparan sulfate improves pre-BCR signaling induced by anti IgM cross-linking [107]. Galectin-1 (GAL1), an S-type lectin which binds β-galactoside glycoconjugates through its carbohydrate recognition domain (CRD), is a ligand for the pre-BCR and binds λ5-EL through direct protein–protein contacts [108,109]. GAL1 is secreted by stromal cells and acts as a docking protein by interacting with both the pre-BCR and glycosylated chains of integrins at the surface of pre-B cells to form a complex lattice [110,111]. Clustering of the pre-BCR is further increased by the interaction of the pre-B cell integrins with their ligands expressed by stromal cells, resulting in pre-BCR signaling. Accordingly, the inactivation of GAL1 expression by stromal cells in vitro and in vivo impairs large pre-BII cell proliferation and differentiation [111]. More recently, we identified GAL1-expressing stromal cells in the BM. Such cells are different from IL7-expressing cells and are not localized in peri-sinusoidal regions [75]. Most importantly, large pre-BII cells are in close contact with these GAL1^+^ cells. While pro-B cells need high levels of IL7 for their proliferation, large pre-BII cells are sensitive to low levels of IL7 [94], suggesting that the transition from the IL7^+^ to the GAL1^+^ stromal cell niche plays an important role in B cell development.

### 4.4. Immature B Cell Niches

The main features at the immature B cell stage are the negative selection of auto-reactive BCR and their egress to the periphery where they complete their maturation. As compared to pro-B and pre-B cells, immature B cells express low levels of CXCR4 and have a decreased capacity to adhere to VCAM1 in a CXCL12-induced manner [91,112]. Moreover, when mice transgenic for a hen egg lysosome (HEL)-specific BCR were treated with HEL to simulate self-antigen engagement, immature B cells upregulated CXCR4, resulting in reduced egress to the periphery. This suggests that immature B cells are retained in the BM if their BCR is auto-reactive. In line with this result, cannabinoid receptor 2 was shown to be involved in the retention of immature B cells in sinusoids and to favor receptor editing [113]. Furthermore, immature B cells are protected from BCR-mediated apoptosis and have the capacity to reinitiate the recombination-activating genes *RAG1* and *RAG2* when incubated with cells of the BM microenvironment expressing DX5 and low levels of Thy1 [114,115]. Altogether, these results show that endothelial and/or stromal cells play an important role in the retention of auto-reactive immature B cells in the BM and in receptor editing.

### 4.5. Recirculating B Cell and Plasma Cell Niches

BM also represents a privileged homing site for plasma cells (PC) and mature B cells. In particular, the BM represents a reservoir for long-lived PC recently requalified as “memory plasma cells” [116]. In addition, the capacity of mature B cells and plasma blasts to home to the BM is likely critical to ensure protection of the hematopoietic system and of maturing immune cells against pathogens. The CXCR4/CXCL12 axis plays a crucial role in the homing of mature B cells (called recirculating B cells in the BM) and plasma blasts, as demonstrated using CXCR4-deficient cells [48,90,117]. Both subsets localize in peri-sinusoidal regions and even in direct contact with CXCLC12-expressing PSS cells in the case of PC [42,118] (Figure 3). Cells of hematopoietic origin are part of the supportive niche and deliver survival cues to PC and recirculating B cells. Dendritic cells (DC) forming clusters in peri-vascular regions are located in close proximity to recirculating B cells [119]. Upon specific depletion of these DC using *CD11c-DTR* transgenic mice, wild-type but not *Bcl2* transgenic recirculating B cells are lost, demonstrating a role for the DC in their survival. Monocytes, eosinophils, and megakaryocytes are also involved in the survival of long-lived PC through the secretion of A proliferation-inducing ligand (APRIL) and IL6 [120,121,122]. Interestingly, monocytes and eosinophils express CXCR4 and the α4β1 integrin, which participate in the positioning of B cells, including PC close to CXCL12-expressing cells [42,91,122]. The complexity of long-lived PC niches, in which hematopoietic cells provide survival signals, while stromal cells represent an anchoring site, is intriguing. Indeed, long-lived PC are sessile and scattered throughout the BM in contact with stromal cells [123]. PC anchoring to stromal cells is likely to occur via the α4β1 and αLβ2 integrins, since treatment with a combination of monoclonal antibodies (mAbs) against these integrins (clone PS/2 and M17/4, respectively) depletes PC from the BM [124]. Whether a unique BM stromal cell subset expresses the different ligands for α4β1 and αLβ2 (fibronectin and VCAM-1 for the former and ICAM-1 for the latter) remains to be demonstrated. However, these results argue for a model in which plasma blasts use CXCL12-driven migration to reach a limited number of niches made of stromal cells and IL6/APRIL-secreting eosinophils, before switching off migration and anchoring to stromal cells. This would be in agreement with studies showing that eosinophil depletion reduces by 70% the number of PC and that generation of new plasma blasts is accompanied by long-lived PC mobilization from the BM [121,125]. Therefore, BM stromal cells not only are able to give direct signals to differentiating hematopoietic cells, but also act as regulators of long-lived PC.

## 5. Concluding Remarks

At the time of stochastic versus instructive models of lymphocyte commitment and differentiation in the early nineties, pioneer studies by Rolink and others clearly established that BM stromal cells play a key role in the generation and regeneration of the B-lymphocyte lineage [126]. On one hand, this prompted many teams to work with co-culture conditions in order to generate large quantities of mature cells starting from few progenitors. On the other hand, this paved the way toward the search for stromal cells supporting long-term hematopoiesis [127]. More recently, and thanks to the use of reporter mice, the progress made in understanding BM organization and BM stromal cell heterogeneity has been tremendous. The influence of the BM microenvironment on pathologies affecting hematopoietic progenitors has benefited from the important advances in normal HSC niche characterization [128,129]. Resistance and relapse in the case of B cell acute lymphoblastic leukemia, the pathological equivalent of differentiating B cells, also concerns a great proportion of patients, most particularly adults. It is now clear that part of the resistance to treatment is related to protective cues transmitted by stromal cells [130]. Therefore, it is now crucial to translate our current understanding of mouse BM organization to human physiological and pathological situations.
